# Evaluation of an Elementary School–based Educational Intervention for Reducing Arsenic Exposure in Bangladesh

**DOI:** 10.1289/ehp.1409462

**Published:** 2015-05-08

**Authors:** Khalid Khan, Ershad Ahmed, Pam Factor-Litvak, Xinhua Liu, Abu B. Siddique, Gail A. Wasserman, Vesna Slavkovich, Diane Levy, Jacob L. Mey, Alexander van Geen, Joseph H. Graziano

**Affiliations:** 1 Department of Environmental Health Sciences, Columbia University Mailman School of Public Health, New York, New York, USA; 2 University of Chicago and Columbia University Arsenic Project Office, Mohakhali, Dhaka, Bangladesh; 3 College of Physicians and Surgeons, Columbia University, New York, New York, USA; 4 New York State Psychiatric Institute, New York, New York, USA; 5 Lamont-Doherty Earth Observatory, Columbia University, New York, New York, USA

## Abstract

**Background:**

Chronic exposure to well water arsenic (As) remains a major rural health challenge in Bangladesh and some other developing countries. Many mitigation programs have been implemented to reduce As exposure, although evaluation studies for these efforts are rare in the literature.

**Objectives:**

In this study we estimated associations between a school-based intervention and various outcome measures of As mitigation.

**Methods:**

We recruited 840 children from 14 elementary schools in Araihazar, Bangladesh. Teachers from 7 schools were trained on an As education curriculum, whereas the remaining 7 schools without any training formed the control group. Surveys, knowledge tests, and well-water testing were conducted on 773 children both at baseline and postintervention follow-up. Urine samples were collected from 210 children from 4 intervention schools and the same number of children from 4 control schools. One low-As (< 10 μg/L) community well in each study village was ensured during an 18-month intervention period.

**Results:**

After adjustment for the availability of low-As wells and other sociodemographic confounders, children receiving the intervention were five times more likely to switch from high- to low-As wells (*p* < 0.001). We also observed a significant decline of urinary arsenic (UAs) (*p* = < 0.001) (estimated β = –214.9; 95% CI: –301.1, –128.7 μg/g creatinine) among the children who were initially drinking from high-As wells (> Bangladesh standard of 50 μg/L) and significantly improved As knowledge attributable to the intervention after controlling for potential confounders.

**Conclusions:**

These findings offer strong evidence that school-based intervention can effectively reduce As exposure in Bangladesh by motivating teachers, children, and parents.

**Citation:**

Khan K, Ahmed E, Factor-Litvak P, Liu X, Siddique AB, Wasserman GA, Slavkovich V, Levy D, Mey JL, van Geen A, Graziano JH. 2015. Evaluation of an elementary school–based educational intervention for reducing arsenic exposure in Bangladesh. Environ Health Perspect 123:1331–1336; http://dx.doi.org/10.1289/ehp.1409462

## Introduction

Arsenic (As) in drinking water is a global environmental health concern because > 200 million people in the world are chronically exposed to As at levels above the World Health Organization (WHO) safety standard of 10 μg/L (Ravenscroft et al. 2009; WHO 2008). Associations of drinking water As with all-cause, as well as chronic, disease mortality have been documented in Bangladesh, one of the most affected countries (Argos et al. 2010; Sohel et al. 2009). Children exposed to As are also at a higher risk of neurodevelopmental deficits and skin lesions (Ahamed et al. 2006; Parvez et al. 2011; Rahman et al. 2010; Wasserman et al. 2004, 2007, 2011). Under the Bangladesh Arsenic Mitigation Water Supply Program (BAMWSP) and related projects, millions of wells have been tested and > 100,000 deep community wells that are low in As have been installed across the country (van Geen et al. 2006). The risks associated with drinking high-As well water have been communicated across 56 districts to raise awareness among rural people (Paul 2004). Agencies such as UNICEF (United Nations Children’s Fund), the Department of Public Health Engineering (DPHE), the Bangladesh governmental institution for provision of drinking water, and a few non-governmental organizations (NGOs) such as NGO Forum and BRAC Bangladesh have developed printed information materials and radio/TV-based public service announcements to educate rural people (Ahamed et al. 2006; UNICEF-Bangladesh 2000). Despite initial success in developing awareness level on As exposure among the villagers, As exposure remains high in many regions across the country (Flanagan et al. 2012; George et al. 2013; van Geen et al. 2014).

A “safe” drinking-water source in rural Bangladesh typically means a low-As well (< 50 μg/L of As, the Bangladesh standard) because other options are likely to be contaminated with waterborne pathogens (Howard et al. 2006). Even when a low-As well is available within walking distance, however, previous studies have shown that a sizeable fraction of neighboring villagers continues to drink from their own high-As well (Chen et al. 2007; George et al. 2012a; Hanchett et al. 2002; Opar et al. 2007). The hypothesis motivating the present study is that a teacher-driven, elementary school–based education program could encourage a larger fraction of the population to switch to low-As wells when paired with well-testing programs and community low-As well installations. Since 2000, the Health Effect of Arsenic Longitudinal Study (HEALS), conducted in Araihazar, Bangladesh, has documented that urinary As (UAs) is a strong predictor of the risk for skin lesions (Ahsan et al. 2000) and that UAs concentration decreases when individuals, in response to a community-based As education program, switch from a high-As to a low-As well (Chen et al. 2007). We built on these findings to test the hypothesis that a targeted educational intervention can maximize the benefits of other mitigation efforts on the exposure of children.

## Methods

**Figure 1:**
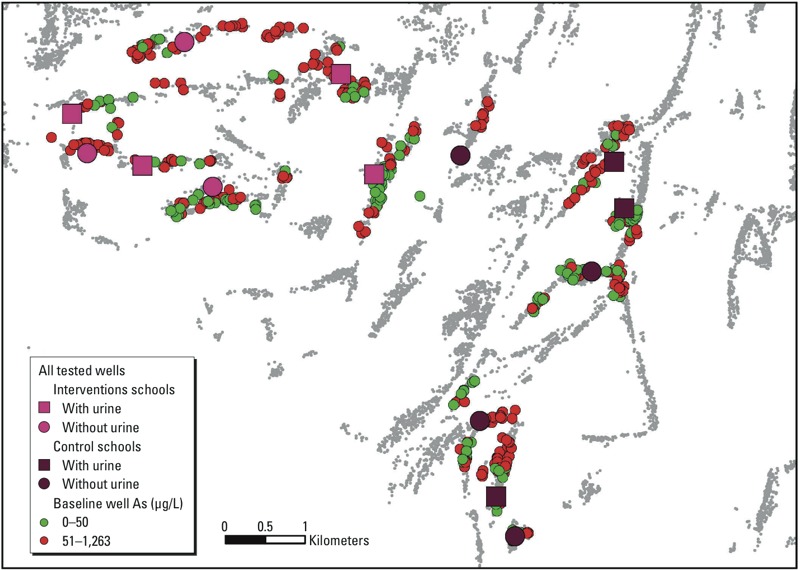
Map of the study area in Araihazar, Bangladesh, showing the locations and status of baseline wells for the children and locations of community wells, which were installed during the intervention period.

*Study area.* The study was conducted in Araihazar, Bangladesh, in villages adjacent to the study area used for the HEALS study (Ahsan et al. 2006). This 183-km^2^ area has 12 unions (the smallest administrative units in Bangladesh), each consisting of 20–30 villages where families rely on a household well to obtain groundwater for drinking and cooking. We report on children who were recruited from 14 elementary schools located in 3 unions (Haizadi, Uchitpur, and Khagkanda) in Araihazar (Figure 1).

*Recruitment.* We initially identified 27 elementary schools in the three unions that were within reasonable travel distance to the HEALS field clinic, of which 14 agreed to participate. Unions were then divided into two clusters: A (Haizadi union) and B (Uchitpur and Khagkanda unions). The two clusters were randomly assigned as intervention (cluster A) and control (cluster B) groups, with seven schools located in each cluster. In this manner, we ensured that intervention and control schools located in these unions remained geographically separated. This cluster assignment aimed to minimize exchange of knowledge across the two groups of children.

From a list of students (*n* = 1,925) provided by the registration and enrollment offices of the 14 participating schools, we randomly selected a cohort of 952 potential participants living in 30 villages. Only one child from each family was included in the cohort. Inclusion criteria restricted enrollment to children 8–11 years of age who attended school regularly in an age-appropriate grade and had no known physical disability. Of these 952, we were unable to find 75, and did not consider 37 others because of age and poor attendance, thus achieving a sample size of 840 with equal numbers for each cluster.

*Procedure.* We secured approval from Institutional Review Boards at Columbia University Medical Center and the Bangladesh Medical Research Council and then obtained written informed consent from parents and schoolteachers, as well as child assent. Between March and October of 2008, we collected baseline sociodemographic information, such as parental education and occupations, home construction characteristics, and availability of television, through a structured parent interview. Well water from participating households and school wells was tested for water As (WAs) on site using the Hach EZ As kit (product no. 2822800) (George et al. 2012b; van Geen et al. 2005) immediately after the parent interview. Samples were also collected from the household wells (i.e., the primary source of drinking water) of all 840 participants for confirmation of field kit results by laboratory analysis. The laboratory results were communicated to the families of the participants and school administrations between May and August of 2009, roughly the mid-point of the intervention period. The field kit correctly determined the status of about 90% of household wells relative to the local (Bangladesh) standard of 50 μg/L WAs.

Both intervention and control schools were randomly divided into two subgroups at baseline; one group provided urine samples and the other did not. Urine samples from 210 intervention children (from a subgroup of four intervention schools) and 210 control children (from a subgroup of four control schools) were then collected. All 840 children took a 15-item As knowledge quiz at baseline.

Our baseline activities were followed by teacher training in small groups at the intervention schools. Together with a “trainer,” who was a project staff member, the teachers developed an As education curriculum for the children, which they implemented in their schools from late 2008 to the middle of 2010. Nineteen deep low-As (< 10 μg/L by laboratory analysis) community wells, including 6 at the schools, were installed by our international NGO partner, WaterAid Bangladesh (WAB 2014), between late 2009 and mid 2010 (Figure 1). This activity ensured the presence of at least one low-As community well in each village, offering all the villagers access to low-As drinking water with WAs concentration below the WHO guideline of 10 μg/L. Based on a subsequent blanket well survey, conducted in 2012–2013, the location of all wells in Araihazar was recorded using hand-held global positioning system receivers (van Geen et al. 2014). Before testing, each household owning a well was asked what it believed the status of its well (with respect to As) to be, and the response was recorded as safe (low As), unsafe (high As), or unknown. Although the proportion of wells of unknown status was high at 52%, the wells believed to be safe in most cases turned out indeed to be safe upon testing relative to the national standard of 50 μg/L (van Geen et al. 2014). Using ArcGIS (ESRI), this information was used to calculate the number of wells believed to be safe within 200 m of each participating household as a proxy for the availability of safe water sources over the course of the school-based intervention. Previous work in Araihazar has shown that 200-m distance is the upper limit for how far households are willing to walk on regular basis to fetch safe water from another well, and that distance to the nearest safe well affects switching (Chen et al. 2007; Opar et al. 2007).

Between March and November of 2010, when the follow-up visits took place, our field staff collected water samples from the wells that were most often used by the families of the participating children, using 20-mL scintillation vials. At the same time, children took the follow-up 15-item quiz (containing the same questions used at baseline); these follow-up visits occurred between 3 and 6 months after the installation of the above-mentioned 19 deep low-As community wells. Follow-up activities also included collection of urine samples from the subset of children from four intervention and four control schools who had provided urine at baseline. We lost 34 intervention and 33 control children at follow-up because their families had moved out of the villages, leaving 773 children with pre- and postintervention information at the end of the study.

*Well water measurement of As.* Well-water samples were collected in 20-mL polyethylene scintillation vials and then acidified with high-purity Optima HCl for at least 48 hr before analysis to ensure re-dissolution of any iron oxides (van Geen et al. 2007). Samples were diluted 1:10 in a solution spiked with 73Ge and 74Ge for internal drift correction and analyzed for As by high-resolution inductively coupled plasma mass spectrometry (Cheng et al. 2004; van Geen et al. 2005). The detection limit of the method was typically 0.2 μg/L. The long-term reproducibility of WAs measurements determined from consistency standards included with each run averaged 4% (1 sigma) in the 40–500 μg/L range. For on-site field-kit assays, we used the two-step Hach EZ As kit. The test involved mixing of prepackaged sulfamic acid and zinc powder with 50 mL of well-water sample. The mixture eventually generated arsine gas (AsH_3_), which is entrained with H_2_ bubbles emanating from the acidified sample and trapped by a strip of paper impregnated with mercuric bromide (van Geen et al. 2005). A previous study showed that this specific procedure could underestimate 50–100 μg/L As range and recommended a modification of the procedure by changing the reaction time from 20 to 40 min (van Geen et al. 2005). At the end of the 40-min reaction time, we observed the intensity of the color of the orange-brown circle on the strip and compared it visually with a reference scale of several colored readings corresponding to As concentrations of 0, 10, 25, 50, 100, 250, and 500 μg/L.

*Urinary measurement of As*. Spot urine samples were collected from the children with the help of a parent in the home. The samples, collected in 50-mL acid-washed polypropylene tubes, were subsequently kept in portable coolers. Within 6 hr of collection, all urine samples were frozen at –20°C, and then shipped to Columbia University on dry ice. UAs concentrations were measured by graphite furnace atomic absorption spectrophotometry using a PerkinElmer Analyst 600 system (Nixon et al. 1991). UAs levels were adjusted for creatinine concentrations and analyzed by a colorimetric method based on Jaffe’s reaction (Heinegård and Tiderström 1973).

*As knowledge assessment.* The same 15-item quiz was administered to participants at both pre- and postintervention phases. Children received 1 point for each correct answer. Points were added to generate a quiz summary score ranging from 0 to 15 for each child. Topics of the knowledge assessment included health effects and disease transmission potential of As, existence of As in environmental media, time span required to produce symptom in As-exposed individuals, types of As-exposure related skin lesions, color codes (red/green) used to mark low- and high-As wells, methods for removal of As from drinking water, interventions for arsenicosis patients, and maintenance of low-As wells.

*Educational intervention materials.* Several publications from the NGO Forum (NGOF 2014) including rhyme and story books and a few posters referring to WAs were identified. Two of these books suitable for elementary school children in Bangladesh were selected for the study. The rhymes and stories presented in these books conveyed information about health effects of As and various mitigation options to reduce As exposure. Four posters, each presenting awareness-building messages on As, were also selected. The posters demonstrated the following messages with pictures: *a*) If arsenicosis persists the patient must drink As-free safe water, eat nutritious foods such as fish, meat, egg, milk, fresh vegetables and fruits, and follow the advice of a physician. *b*) A person should report to a nearby health center if his/her palms and soles become rough and brown or black spots appear in the skin. *c*) Arsenicosis is not a communicable or hereditary disease. *d*) Arsenicosis cannot be transmitted. NGO Forum gave us permission to distribute these materials in the participating schools.

*Training for teachers.* On the first day of the 2-day-long teacher training, various concepts behind groundwater As problems were introduced. The second day focused on practical mitigation options. Teachers also learned how to organize awareness-building and engaging activities and interschool competitions to motivate children and their families. Finally, intervention materials were presented, and strategies needed for optimal utilization of these resources were discussed.

*As education in schools.* Each intervention school received four sets of bound posters to be hung in the classrooms, and 8- to 11-year-old children (typically 3rd to 5th graders) received free copies of rhyme and story books. The teachers were advised to spend three 15-min sessions per week to educate children about As and were advised to organize fieldwork in the communities bimonthly through a rally. Intra- and interschool “arsenic art” competitions on As-related subjects were also arranged once a year.

*Deep community well installation.* Our initial goal was to install or ensure the presence of a low-As well in each village and school by the time of the launch of the classroom education effort. However, the installation plan could not be implemented by WAB until December 2009, roughly the mid-point of the As-education program. Eleven of 30 study villages where the families of our participants resided had at least one low-As community well at baseline. Eight of 14 schools involved in our study also had low-As wells at baseline. Therefore, we decided to provide new community wells to the remaining 19 villages and 6 schools (2 intervention and 4 controls). WAB, on consultation with the community leaders, selected the sites for installing one new low-As (< 10 μg/L) well in each of these 19 villages and 6 participating schools during the intervention period. The depth of these new village and school wells (also known as community wells) ranged from 650 to 780 ft, and water pumped from these wells contained between 0.2 and 1.5 μg/L of As.

*Statistical analyses.* Summary statistics were calculated to describe the sample characteristics. Chi-square and *t*-tests were used to detect differences between the intervention and control groups for categorical variables and continuous variables (transformed to improve normality if necessary), respectively. Paired *t*-tests were used to compare pre- and postintervention quiz (knowledge assessment) scores. The outcome variables of interest were the changes from pre- to postintervention in *a*) well-switching behaviors from high-As baseline wells to low-As wells, *b*) the change in UAsCr (UAs adjusted for creatinine), and *c*) changes in scores of the quiz on As knowledge. We used logistic models to examine differences between intervention and control groups in various well-switching behaviors such as *a*) switching to a new well versus continuing to use the same well (all participants), *b*) switching to a low-As well versus switching to a high-As well among all those who switched wells, and *c*) switching to a low-As well versus switching to a high-As well among those who switched wells and had a high-As well at baseline. We used linear models for group difference in the changes of UAsCr and quiz scores controlling for potential confounding variables. On the basis of a literature review, we identified some potential confounding variables that could differ between the intervention and control groups. We evaluated whether inclusion of potential confounding variables changed the estimated regression coefficient relating group (intervention vs. control) to the outcome by > 0.5 standard errors. Variables that met this criterion—such as maternal and paternal education (dichotomous; with vs. without education), sex (dichotomous; boys vs. girls), television in house (dichotomous; with vs. without TV), school grade (three categories), house construction (dichotomous; materials used in construction of house-wall), and urine sampling status (dichotomous; if urine sample was collected from the subject)—were included in the final regression models (indicated by model A). For well-switching behavior and change in UAs/g Cr from baseline to follow-up, we additionally controlled for the number of low-As wells available within 200 m of the household, which was used as a continuous variable (indicated by model B). For model B, we considered the subset of children who were drinking from wells containing levels of As higher than the Bangladesh standard (> 50 μg/L) at baseline. Power calculations for this study were based on two-sided chi-square test with significance level of 0.05. Given 40% of well switching in the control group, the sample size of 400 (200 per group) would enable us to detect a well-switching rate of 50% in our intervention group with power of 83%. These meaningful rates were selected on the basis of one of our previous epidemiological studies in the same geographical location (Chen et al. 2007).

## Results

*Sample characteristics.* We first compared the two groups of children who completed the study (Table 1). No significant differences between the intervention and control groups were found for child’s body mass index, sex, school grade, or paternal education levels. Children in the intervention group were more likely to have better (i.e., concrete) house construction, more access to television in house, and better maternal education (*p* < 0.05) and had slightly higher WAs at baseline than controls (127.0 vs. 111.7 μg/L; *p* = 0.16). The difference in WAs between the two groups is consistent with significantly higher UAs in the intervention group (433 vs. 289 mg/g creatinine; *p* < 0.001).

**Table 1:** Sociodemographic and exposure characteristics of the children from the intervention and control groups.

Variable	Intervention (*n *= 386)		Control (*n *= 387)		*p*-Value (*t*-test/ χ^2 ^test)^*c*^
	Mean ± SD or *n* (%)^*a*^	Range^*b*^	Mean ± SD or *n* (%)^*a*^	Range^*b*^	
Age (years)	9.2 ± 0.8	8.0–10.9	9.5 ± 0.8	8.0–11.0	0.01
Body mass index (kg/m^2^)	14.2 ± 1.2	10.9–19.1	14.2 ± 1.2	10.8–18.7	0.61
Head circumference (cm)	49.5 ± 1.5	45.3–55.0	49.1 ± 1.4	45.4–53.1	0.01
Baseline WAs (μg/L)	127.0 ± 147.5	0.1–853.7	111.7 ± 151.1	0.09–1263.2	0.16
Follow-up WAs (μg/L)	43.9 ± 92.0	0.04–880.5	80.9 ± 97.4	0.04–960.8	< 0.001
Baseline UAs (μg/L)^*d*^	171.1 ± 169.2	7.0–910.0	112.6 ± 81.3	6.0–462.0	< 0.001
Follow-up UAs (μg/L)^*d*^	182.2 ± 206.7	6.0–1465.0	145.8 ± 107.1	12.0–772.0	0.03
Baseline UCr (mg/dL)^*d*^	45.5 ± 34.8	4.5–251.3	45.3 ± 33.8	4.1–185.3	0.96
Follow-up UCr (mg/dL)^*d*^	63.4 ± 51.4	6.9–506.0	66.9 ± 43.1	5.5–274.2	0.47
Baseline UAs (mg/g creatinine)^*d*^	433.0 ± 372.0	47.4–2589.7	289.4 ± 162.0	50.2–1042.0	< 0.001
Follow-up UAs (mg/g creatinine)^*d*^	321.6 ± 292.7	13.8–1880.6	248.8 ± 171.8	51.1–1181.8	0.003
No. of wells perceived as “safe” (low As) within 200 m of the households^*e*^	9.8 ± 10.6	4.0–67.0	19.9 ± 10.0	0.0–51.0	< 0.001
High-As wells (> 50 μg/L) at baseline	220 (57.0)		260 (67.2)		0.004
High-As wells (> 50 μg/L) at follow-up	82 (21.2)		211 (54.5)		< 0.001
Boys	173 (44.8)		183 (47.3)		0.49
Television in house	199 (51.6)		154 (39.8)		0.001
School grade
Up to 2nd grade	117 (30.3)		142 (36.7)		0.17
3rd grade	191 (49.5)		172 (44.4)
4th grade	78 (20.2)		73 (18.9)
House construction
Concrete	32 (8.3)		18 (4.7)		0.04
Corrugate/biomass/mud	354 (91.7)		369 (95.3)
Paternal education
Received formal education	193 (50.0)		208 (53.8)		0.30
Without formal education	193 (50.0)		179 (46.2)
Maternal education
Received formal education	202 (52.3)		165 (42.6)		0.007
Without formal education	184 (47.7)		222 (57.4)
^***a***^For continuous variables mean (± SD) and for categorical variables *n* (%) are reported. ^***b***^Maximum and minimum values. ^***c***^To report group differences for continuous variables, *t*-test is used; for categorical variables, chi-square test is used. ^***d***^Urine samples were collected from 194 intervention children and 197 follow-up children.^***e***^Number of wells perceived as “safe” (low As) was available for 220 intervention and 260 control children.					

*Well-switching behavior.* At baseline, 57% (220 of 386) and 67% (260 of 387) of participants in the intervention and control groups, respectively, were drinking from high-As wells (i.e., > 50 μg/L, the Bangladesh standard). Of these, 68% of intervention children (149/220) and 23% of control children (59/260) switched to low-As wells during the intervention period. After adjustment for sociodemographic variables, among children whose families used a high-As well at baseline and who switched wells before follow-up, families of the children in the intervention group were 3.4 times more likely to switch from a high- to a low-As well (vs. from a high-As well to another high-As well) than control families [95% confidence interval (CI): 1.8, 6.3] (Table 2). The average number of wells believed (by participants families) to be safe within 200 m of each participating household turned out to be significantly higher for the control group (mean ± SD, 19.9 ± 10.0) compared with the intervention group (mean ± SD, 9.8 ± 10.6). The difference is significant (*p* < 0.001) and indicates more opportunities for switching to a low-As source within the control group. After additionally controlling for the number of wells perceived to be low As within 200 m of the house the odds ratio (OR) for the likelihood of switching from a high-As well to a low-As well among those who had a high-As well at baseline and who switched wells during follow-up increased to 5.3 (95% CI: 2.5, 11.5).

**Table 2:** Odds ratio (95% CI) of well-switching behaviors comparing intervention to control group.

Outcome	Model A^*a*^			Model B^*b*^		
	Intervention group (*n*)	Control group (*n*)	OR (95% CI)	Intervention group (*n*)	Control group (*n*)	OR (95% CI)
Any well switching vs. no switching
Switched to another well	241	148	2.6 (1.9, 3.4)*	179	111	9.1 (5.2, 15.9)*
Did not switch	145	239		41	149
Switched to low-As well vs. switched to high-As well
Switched to a low-As well	200	86	3.1 (1.9, 5.3)*	149	59	5.3 (2.5, 11.5)*
Switched to high-As well	41	62		30	52
Switched from high-As to low-As well vs. switched from high-As to high-As well
Switched from high- to low-As well	149	59	3.4 (1.8, 6.3)*	149	59	5.3 (2.5, 11.5)*
Switched from high- to high-As well	30	52		30	52
^***a***^Model adjusted for maternal and paternal education, sex, television in house, school grade, house construction, and urine sampling status (whether the child was included in the subgroup that provided urine sample). ^***b***^This model examined the subset of 480 children (220 intervention and 260 control) who had high As (> 50 μg/L) at baseline; model B was additionally adjusted for the number of wells perceived to be low As within 200 m of the house among the children who had high-As wells at baseline. *p < 0.01.						

**Figure 2:**
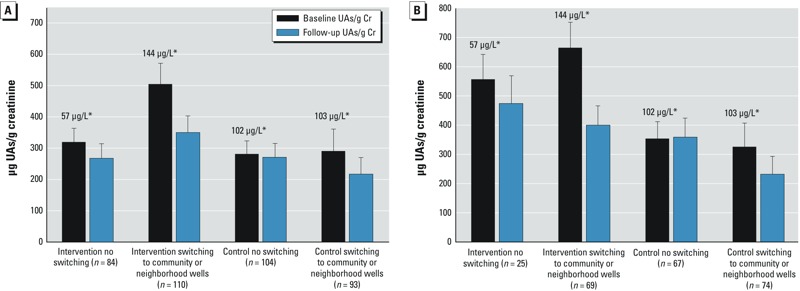
(*A*) Mean urinary creatinine-adjusted As levels for children in intervention and control groups by their switching status after accounting for maternal education, paternal education, sex, television in house, house construction, and school grade. (*B*) Model B examined the subset of 235 children (94 intervention and 141 control) who had high As (> 50 μg/L) at baseline, and additionally adjusted for the number of wells perceived to be low As within 200 m of the house. *Values above the bars are average baseline well As concentrations. Error bars indicate upper bound of 95% CI.

*Intervention and change of UAs.* The intervention group experienced a greater decline in Cr-adjusted UAs than did the control group (estimated group difference in mean change β = –70.8; 95% CI: –122.2, –19.4 μg/g Cr) after adjusting for the sociodemographic confounders. After additionally accounting for the number of wells perceived to be low As within 200 m of the house in the subset of children who had high-As wells at baseline, we observed a higher magnitude of UAs/gCr reduction attributable to this intervention (β = –214.9; 95% CI: –301.1, –128.7 μg/g Cr). We observed that the mean UAs/gCr substantially declined only among the subgroups of intervention and control children who were able to switch wells during intervention period after accounting for the sociodemographic covariates (Figure 2). Also, we observed that the decline in UAs/gCr among the intervention children was dependent on higher baseline water WAs concentrations; that is, none of the baseline categories other than intervention groups with WAs of 101–200 μg/L and > 200 μg/L showed significant declines (Table 3). Switching from the baseline household well to a low-As well (deep community or neighbor’s low-As well) resulted in a significant (*p* < 0.05) decline in UAs in both of these groups.

**Table 3:** Change in creatinine-adjusted urinary arsenic in intervention and control children by baseline WAs categories.

Group	Baseline water As (μg/L)	*n*	Unadjusted model		Model A^*a*^		Model B^*b*^	
			Change in mean μg UAs/g Cr^*c*^ [β (95% CI)]	*p*‑Value	Change in mean μg UAs/g Cr^*c*^ [β (95% CI)]	*p*‑Value	Change in mean μg UAs/g Cr^*c*^ [β (95% CI)]	*p*‑Value
Intervention	0–50	100	–10.2 (–68.7, 48.4)	0.73	–20.8 (–150.9, 109.4)	0.75
	51–100	26	–22.4 (–151.3, 106.6)	0.73	–34.1 (–165.7, 97.5)	0.60	–138.4 (–995.6, 718.9)	0.74
	101–200	30	–162.0 (–283.9, –40.1)	0.001	–166.1 (–291.5, –40.7)	0.001	–324.2 (–649.0, 0.66)	0.05
	> 200	38	–373.7 (–485.3, –262.1)	< 0.001	–380.8 (–495.6, –266.0)	< 0.001	–544.4 (–803.0, –225.8)	< 0.001
Control	0–50	56	–29.3 (–72.9, 14.3)	0.18	–51.6 (–127.0, 23.9)	0.18		
	51–100	69	10.3 (–48.4, 68.9)	0.73	8.8 (–51.0, 68.7)	0.77	–215.2 (–466.4, 36.0)	0.10
	101–200	55	–31.2 (–93.1, 30.7)	0.32	–33.7 (–96.5, 29.1)	0.29	–0.1 (–92.5, 92.4)	0.99
	> 200	17	–71.6 (–161.9, 18.7)	0.12	–76.2 (–170.9, 18.5)	0.11	–81.5 (–197.8, 34.8)	0.17
^***a***^Model adjusted for maternal and paternal education, sex, television in house, school grade, and house construction. ^***b***^This model examined the subset of 235 children (94 intervention and 141 control) who had high As (> 50 μg/L) at baseline; model B was additionally adjusted for the number of wells perceived to be low As within 200 m of the house among the children who had high-As wells at baseline. ^***c***^Change in UAs/g Cr from baseline to follow-up.								

*Intervention and change of As knowledge.* On average, intervention and control children received summary scores on the baseline quiz of 9.3 and 8.4 points (of 15), respectively. From the baseline to follow-up, intervention and control quiz summary scores improved by 5.3 and 1.5 points, respectively (*p* < 0.001 for group difference). Adjustment for school grade, maternal and paternal education, sex, house construction (as a proxy for wealth), and access to television at home did not materially change these findings (data not shown).

## Discussion

A combination of approaches (e.g., education, well testing, community well installation) have been recommended in several studies to maximize the benefit of As mitigation (Chen et al. 2007; George et al. 2012a; Opar et al. 2007). Our intervention is unique in that we have not only combined these approaches but also enhanced community engagement by enabling the schoolteachers to become educators concerning arsenic mitigation. During the intervention period, the teachers conveyed information regarding the benefit of switching from a high- to a low-As well to the parents, more specifically to the mothers of the participants. In addition, children were asked by the teachers to do the same. In Bangladesh, mothers actually fetch the household water; therefore, it is likely that the entire family can benefit from such an intervention.

Use of multiple outcomes—such as well-switching behavior, a biomarker of exposure (UAs), and change in knowledge about As to evaluate the efficacy of an intervention program—strengthens our findings. The findings on well-switching behavior are consistent with those of previous studies in the same geographic region. These studies reported switching from high-As (> 50 μg/L) to low-As well for 58% (Chen et al. 2007) and 65% (Opar et al. 2007) of the participants in response to in-person communication of test results, health education, well labeling, and low-As deep well installation. In our study, 68% of the participants of the intervention group who had high As at pre-intervention ended up switching to low-As wells after intervention as opposed to 23% of the controls showing the same type of well-switching behavior.

At baseline, children and their families were informed about the levels of WAs in their household wells (field kit assessment), and later they received more accurate laboratory results. Information about their baseline well status encouraged the families to move from a high- to a low-As well producing a substantial decline in As exposure among the children of both intervention and control groups (Figure 2) a finding that is consistent with another community-based study in Araihazar (Chen et al. 2007). We observed a dose–response relationship between pre-intervention WAs status and change in UAs among the intervention participants (Table 3). There was no significant change in UAs in children who had baseline WAs either at “low As” level (< 50 μg/L) or close to “low As” level (51–100 μg/L). We speculate that many families, who had baseline well As concentrations between 51 and 100 μg/L, a range close to the Bangladesh standard, were reluctant to make a switching attempt especially when the number of low-As well options were limited.

Our findings indicate that despite the availability of a lower number of low-As wells within 200 m of the intervention participants’ homes, the reduction of As exposure was substantially higher in this group. The largest declines (60% in intervention and 40% in control) were observed in children who switched from household wells to newly installed low-As community wells, though the number of children whose families switched to the new wells was relatively small (15 intervention and 10 control families). This finding underlines the importance of providing public sources of low-As water by installing deep community wells across As-affected villages in Bangladesh. In addition, installation of six new school wells [two intervention and four control schools (Figure 1)] might have contributed to a decline in UAs across all subgroups. These findings reinforce the need for an educational program to maximize reduction of As exposures in rural communities.

## Conclusions

School-based As education has several advantages over conventional community-based education in developing countries like Bangladesh. First, it can effectively use local resources (e.g., trained schoolteachers who often live in villages close to the schools) rather than short-term project-based field staff, thus reducing the cost and ensuring sustainability. Second, by educating thousands of children, the knowledge can be widely disseminated within a short time. Third, the teachers in rural communities are among the most respected professionals and are often consulted directly by poor, illiterate people on various issues. Teachers trained on the As issue could take advantage of this advisory role to motivate people to drink from low-As wells.

In Bangladesh, well water contributes the major portion of As consumed by millions of rural people. The present study indicates that the education of elementary school children about the hazards of As consumption can serve as part of an integrated strategy to reduce As exposure in affected regions.
